# Socio-Economic Status Determines Risk of Receptive Syringe Sharing Behaviors among Iranian Drug Injectors; A National Study

**DOI:** 10.3389/fpsyt.2014.00194

**Published:** 2015-03-23

**Authors:** Shervin Assari, Khodabakhsh Ahmadi, Majid Rezazade

**Affiliations:** ^1^Department of Psychiatry, School of Medicine, University of Michigan, Ann Arbor, MI, USA; ^2^Center for Research on Ethnicity, Culture, and Health (CRECH), School of Public Health, University of Michigan, Ann Arbor, MI, USA; ^3^Social Determinants of Health Research Center, University of Social Welfare and Rehabilitation, Tehran, Iran; ^4^Behavioral Sciences Research Center, Baqiyatallah University of Medical Sciences, Tehran, Iran; ^5^AIDS Prevention and Control Committee of Welfare Organization State, Tehran, Iran

**Keywords:** injection drug users, HIV, needle and syringe sharing, socio-demographics, Iran

## Abstract

**Background:** Although needle and syringe sharing is one of the main routs of transmission of HIV in several countries in the middle east, very little is known about how socio-economic status of injecting drug users (IDUs) is linked to the receptive syringe sharing behaviors in these countries.

**Aim:** To study socio-economic correlates of receptive needle and syringe sharing among IDUs in Iran.

**Methods:** The study used data from the Unhide Risk Study, a national survey of IDUs. This study sampled 636 IDUs (91% male) via snowball sampling from eight provinces in Iran in 2009. Socio-demographic and drug use characteristics were collected. We used a logistic regression to determine factors associated with receptive needle and syringe sharing during the past 6 months.

**Results:** From 636 IDUs enrolled in this study, 68% (*n* = 434) reported receptive needle and syringe sharing behaviors in the past 6 months. Odds of receptive needle and syringe sharing in the past 6 months was lower among IDUs who were male [odds ratios (OR) = 0.29, 95% confidence interval (CI) = 0.12–0.70], had higher education (OR = 0.74, 95% CI = 0.64–0.86) but higher among those who were unemployed (OR = 4.05, 95% CI = 1.50–10.94), and were single (OR = 1.47, 95% CI = 1.02–2.11).

**Conclusion:** This study presented factors associated with risk of receptive needle and syringe sharing among Iranian IDUs. This information may be used for HIV prevention and harm reduction purposes. Socio-economic status of Iranian IDUs may be closely linked to high-risk injecting behaviors among them.

## Introduction

Increasing trend of HIV and AIDS is a global health challenge (1). In several Middle Eastern countries, this epidemic is closely attached to the pattern of high-risk injecting behaviors among injecting drug users (IDUs) (2). In Iran, at least 6 of 10 HIV positive cases and 8 of 10 AIDS cases occur among IDUs (3). In Iran, needle-sharing behaviors have been consistently shown to be a main route of HIV transmission (4–8).

The Iranian Ministry of Health has recently announced an estimated figure of 200,000 IDUs in Iran (9). Although Iran has a number of harm reduction programs for tackling epidemics of HIV and other blood born infections among IDUs and other high-risk groups (6, 10–12), prevalence of HIV is increasing rapidly among infections among IDUs in Iran (6). High prevalence of HIV (10–20%) and HCV (30–50%) among Iranian drug injectors is well known (13–18). High prevalence of HIV and other blood born infections among Iranian IDUs have been attributed to their high rate risk-behaviors such as shared injection and needle sharing (3, 6–8, 19, 20).

Globally, rate of syringe sharing varies based on geographic regions. Shared injecting behaviors have been reported to be from 25% in Canada (21) to 80% in Mexico (19). Prevalence of shared injection among Iranian IDUs differs widely from one study to another. Lifetime needle and syringe sharing among IDUs was one-third among IDUs in 2009 (19), half in one national survey in 1999 (20), and finally two-thirds in another study published in 2006 (5).

A wide range of socio-economic factors may be associated with high-risk injecting behaviors among IDUs. Socio-demographic characteristics such as gender (19), marital/partnership status (22), employment (19), economic status (19), living condition (19), and place of residence (23) may influence pattern of risk-behaviors among IDUs. In addition to the socio-economic factors, drug use characteristics such as years passed from first drug injection (24), frequency (21) and setting (25, 26) of injection, and poly drug use (21) may influences high-risk injections of IDUs. HIV sero-status and HIV knowledge may also contribute to high-risk injections of IDUs (22).

In Iran, little is known about the socio-economic and drug-related predictors of risk-behaviors among Iranian IDUs (3, 6, 7). A recent report suggested that female gender [odds ratios (OR) = 2.68] and being jobless (OR = 1.87) were associated with higher risk of shared injection, while being alone at most injections (OR = 0.51) was associated with lower risk of needle and syringe sharing (19). That study is, however, limited for two reasons. First, the study entered other high-risk-behaviors to the list of predictors of lifetime needle sharing in the model. Including other risk factors may result in over adjustment or at least un-necessary adjustment (27–29). Secondly, as outcome is lifetime needle and syringe sharing history (19), we do not have information on receptive sharing behaviors.

To set harm reduction policies, policy makers need information on rate and associated factors of risky behavior. This study is thus aimed to determine the rate and predictors of receptive needle and syringe sharing among Iranian IDUs.

## Materials and Methods

### Design and setting

With a cross-sectional design, data came from Unhide Risk, a national survey of IDUs. This study sampled 636 IDUs (91% male) via snow ball sampling from eight provinces in Iran in 2009. Some other manuscripts have been published from this database (30–32).

### Codes of ethics

The study was approved by the ethical review committee of the Baqiyatallah University of Medical Sciences. Informed consent was obtained from all the participants after they had been verbally reassured that the information would be kept confidential, especially from the correctional system. All questionnaires were anonymous. No financial incentives were given to the participants, however, all participants received HIV education, and also free syringes and condoms.

### Process

Data were collected via structured interviews, using a questionnaire. Interviews were performed by research area specialists who had received trainings through workshops held in Tehran, Iran. Each interview took up to 60 min.

Data were collected using paper-based questionnaires, which included: (1) socio-demographic, (2) family data, (3) history of childhood trauma, (4) HIV attitude, (5) HIV knowledge, (6) Injection Behavior, (7) sexual behavior, and (8) HIV test.

### Outcome

Receptive needle and syringe sharing during the past 6 months was measured using these two questions: (A) During the last month, how many times did you inject drugs using shared syringe/needle? Answers included (0) never, (1) only 1–3 times, (2) once a month, (3) 2–3 times a month, (4) 1 time per week, (5) 2–6 times a week, (6) 1 time per day, (7) 2–3 times a day, and (8) 4 times or more a day. (B) During the past 6 months, provided that using shared syringes, usually how many people had injected by that used syringe, before your injection? Answer of 1 or more to both above questions were considered as receptive needle sharing. The 6-month interval has been used for assessing needle sharing, previously (22, 33).

### Statistical analysis

The data obtained in the Statistical Package for the Social Sciences 17 (SPSS Inc, IL, USA) for Windows. In addition to descriptive statistics that included mean and standard deviation (SD) and also median and first and third quartile (25th and 75th percentiles). For bivariate analysis, independent samples *t*-test, and chi-square tests were used. Multivariate stepwise logistic regression was used to determine the predictors of receptive syringe and needle sharing. ORs and 95% Confidence Interval (CI) were reported. *P* value <0.05 was considered significant.

## Results

All 636 participants were street-based IDUs, 71.5% of whom were daily injectors (*n* = 455). 91.1% were male, 57.8% were single, 59.3% were employed, and only 8.3% were illiterate.

Mean age of the IDUs was 31.5 ± 7.8 year. Details of baseline data are presented in Table 1.

**Table 1:** **Socio-demographic and injection frequency among Iranian IDUs (*n* = 636)**.

Characteristics	*n*	%
**Socio-demographic data**
Gender
Male	581	91.1
Female	44	6.9
Missing	13	2.0
Marital status
Single	369	57.8
Married	262	41.1
Missing	7	1.1
Educational level
Illiterate	53	8.3
Primary	140	21.9
Guidance school	232	36.4
High school	95	14.9
High school diploma	90	14.1
University degree	21	3.5
Missing	6	0.9
Living status
Alone	102	16.0
With someone	531	83.2
Missing	5	0.8
Housing
Live in others house	335	52.5
Live in own/rental house	273	42.8
Missing	30	4.7
Employment
Jobless	234	36.7
Employed	378	59.3
Missing	26	4.1
Injection frequency
2–3 times a month	62	9.7
Once a week	53	8.3
2–6 times a week	67	10.5
Once a day	131	20.5
2–3 times a day	198	31.0
4 times or more a day	127	19.9

### Receptive needle and syringe sharing behaviors

Two IDUs (0.3%) did not answer our questions regarding the receptive sharing, and 68% of IDUs (*n* = 434) reported receptive needle and syringe sharing.

Using the Pearson Chi-square test for bivariate analysis showed that being female (OR = 1.07, 95% CI = 1.02–1.11), experience of abuse during childhood (OR = 1.41, 95% CI = 1.23–1.61), and unemployment (OR = 1.05, 95% CI = 1.01–1.08) were associated with higher likelihood of receptive needle and syringe sharing. There was not a significant association between received sharing and living alone or being illiterate (*p* > 0.05 for all) (Table 2).

**Table 2:** **Comparison of socio-demographic and injection frequency among Iranian injection drug users with and without receptive syringe sharing (*n* = 636)**.

	Receptive syringe sharing		Sig.^a^
	Yes *N* = 434	No *N* = 202	
Female	38 (9.0)	6 (3.0)	0.006
Single	261 (61.0)	108 (53.7)	0.085
Unemployed	30 (6.9)	5 (2.5)	0.022
Lived alone	74 (17.1)	28 (13.9)	0.308
Positive history of childhood abuse	216 (51.1)	62 (31.0)	<0.001
Daily injector	319 (73.5)	136 (67.3)	0.108
Illiterate	45 (10.4)	8 (4.0)	0.821

*^a^Chi-square test was employed*.

*Outcome was missed in two participants*.

Based on the independent samples *t*-test, mean age was not significantly different between those with (31.4 ± 7.9 years) and without (31.6 ± 7.8 years) received needle sharing (*p* = 0.75).

Logistic regression showed that the likelihood of receptive needle and syringe sharing in that past 6 months was positively associated with being jobless (OR = 4.05, 95% CI = 1.50–10.94), being single (OR = 1.47, 95% CI = 1.02–2.11), and negatively associated with male gender (OR = 0.29, 95% CI = 0.12–0.70), and having a higher educational level (OR = 0.74, 95% CI = 0.64–0.86) (Table 3).

**Table 3:** **Summary of findings of binary logistic regression on socio-economic and drug-related factors associated with receptive syringe sharing among injection drug users (*n* = 636)**.

Characteristics	Exp (B)	95% C.I. for Exp (B)	
		Lower	Upper
Gender (male)	0.29	0.12	0.70
Marital status (single)	1.47	1.02	2.11
Higher educational level	0.74	0.64	0.86
Unemployment	4.05	1.50	10.94

*Variable(s) entered on this model included age, gender, marital status, educational level, unemployment, living alone, injection frequency, and history of childhood abuse*.

## Discussion

According to the current study, in Iran, about two of three IDUs report receptive needle and syringe sharing. Receptive needle and syringe sharing seems to be higher among female, unemployed, single, and less educated IDUs.

The rate reported in this study seems alarming and higher than some previously reported rates (19, 20). Our report is very similar to the rate reported by Day et al. on the patterns of drug use among a sample of drug users and IDUs attending a general practice in Iran in 2006 (5). That report however was a single center, in clinical setting, reporting shared injection among IDUs (*n* = 292) who were limited from a clinical setting in the capital of Iran. Our rate is, however, only receptive syringe sharing among a community sample of IDUs (*n* = 636) enrolled from eight provinces and has sampled setting.

There is scarce data on the rate, reasons, risk factors and consequences of needle, and syringe sharing among Iranian IDUs. Based on one study, half of the IDUs had shared injection paraphernalia 1–2 months prior to interview. Sharing in prisons was stated to be the rule. Reasons for sharing were indicated as difficulty of purchasing syringes in inaccessible hours, hesitance of pharmacists to sell syringes and also financial strains. The main reason for avoiding sharing among IDUs was the fear of acquiring HIV (34).

A qualitative study of IDUs in Iran reported a possible reduction in the needle sharing. That study however showed that IDUs continue to share drugs in locations of group injection, and in states of withdrawal or severe addiction. That qualitative study stated being homeless, female, and young age as possible factors that may increase the risks for HIV among IDUs. System-wise barriers to harm reduction listed by participants included the cost or stigma of purchasing needles from pharmacies, over-burdened clinics, irregular enforcement of laws protecting IDU, and lack of efforts to address the sexual risks of IDU (8).

In a recent national study on more than 2,000 IDUs in Iran in 2007, lifetime needle and syringe sharing was associated with female gender, being jobless, having illegal income, and history of drug use by family members (35). The first two risk factors reported by that study was also associated with a higher likelihood of receptive syringe sharing among our sample. The outcome, was, however, different in the two studies.

Our study showed a higher likelihood of receptive needle and syringe sharing among female IDUs. Gender has been reported as a factor associated with sharing behaviors in both Iran (35) and in other countries (36, 37). A qualitative study of Iranian female IDUs reported sharing syringes as a typical behavior of IDUs (6). As a result, needle exchange programs need to be sensitive to gender of the target IDU population (38).

Based on the Theory of Gender and Power, the sexual division of labor, the sexual division of power, and the structure of cathexis are the main structures that influence distribution of risk based on gender in a society. Based on this theory, which was developed by Connell in 1987, major gender differences in employment, income, and education may contribute to power imbalances and subordination of women in many aspects in most societies. Due to such inequalities, women are more frequently exposed to risk factors that ultimately influence their well-being (39).

In our study, compared to those with a job, unemployed IDUs were at a higher risk of receptive needle and syringe sharing. Review of literature shows a link between unemployment of IDUs and needle and syringe sharing behavior (40, 41). This might be a direct result of financial strains of unemployment, or a more complex pathway, such as network or associated traits.

In Iran, evidences have shown that access to a needle and syringe program will reduce the needle and syringe sharing practices of the IDUs (8). A quantitative study with cross-sectional design reported that shared use of needle/syringe in the past month was significantly lower among IDUs who received estimated ≥7 syringes per week than those who did not (OR = 14.36), and the findings suggest that the outreach program in Tehran has reduced direct sharing among those who received more than several needles/syringes from the program (42). Another quantitative study in 2005, on 419 street-based IDUs in Tehran compared self-reported needle and syringe access and use between IDUs from a neighborhood with an active NSP to IDUs from a neighborhood without such an intervention. A significantly smaller proportion of IDUs from the former neighborhood reported having used a shared needle/syringe over a 1-month period (21.0%) compared to IDUs from the latter neighborhood (39.9%) (OR = 0.24). That study documented a success of the NSP programs in Iran in reducing needle and syringe sharing practices among IDUs (8).

There are some limitations to this study. First, cross-sectional design of the study limits any causal inference. Future research should go beyond cross-sectional studies and test direction of the associations reported in this study. We did not have data on social context and social network of the IDUs. We also did not enter access to needle exchange programs or HIV education programs to our study. The study relies on participants’ self-report data, which may be affected by response bias. Thus we cannot rule out the possibility of underreported shared injection due to social desirability of the IDUs (43). As samples were not random, findings are not representative of all IDUs.

## Conclusion

Intervention programs that target to reduce HIV risk through promotion of safe injection among Iranian IDUs should consider socio-economic characteristics of their target populations. Such programs may need to enhance enrolment of female, unemployed, single, and less educated IDUs who are at highest risk of receptive needle-sharing behaviors.

## Conflict of Interest Statement

The reviewer Masoumeh Dejman declares that, despite having collaborated with author Shervin Assari, the review process was handled objectively and no conflict of interest exists. The authors declare that the research was conducted in the absence of any commercial or financial relationships that could be construed as a potential conflict of interest.
